# Critical Care Management for Novel 2019 SARS-CoV-2 and HCoV-NL63 Coinfection in a Young Immunocompromised Patient: A Chicago Experience

**DOI:** 10.1155/2020/8877641

**Published:** 2020-07-31

**Authors:** Alejandro Sanchez-Nadales, Miguel Treminio-Quezada, Hasan Abad, Jessica Navarro-Motta, Pamela Contreras-Chavez, Anil Kachru, Chae Chu

**Affiliations:** ^1^Department of Medicine, Advocate Illinois Masonic Medical Center, Chicago, IL 60657, USA; ^2^Department of Pulmonology and Critical Care Medicine, Advocate Illinois Masonic Medical Center, Chicago, IL 60657, USA

## Abstract

**Background:**

SARS-CoV-2 is a newly emerged virus that has spread rapidly, exhibiting tremendous morbidity and mortality. Some potential pharmaceutical targets have been identified but are still lacking proper validation. *Case Presentation*. We describe the case of a young, immunosuppressed and critically ill patient with previous Influenza B infection, requiring extracorporeal membrane oxygenation, which was then followed, in the succeeding months, by SARS-CoV-2 infection complicated by severe adult respiratory distress syndrome. Her clinical course exhibited complications, including pulmonary embolism, acute kidney injury, pneumothorax, pneumomediastinum, multiple cardiac arrests, and eventually death.

**Conclusion:**

Coinfection with other respiratory pathogens and opportunistic infections are possible.

## 1. Introduction

The Severe Acute Respiratory Syndrome Coronavirus 2 (SARS-CoV-2) is a newly emerged virus that first appeared in December 2019. This pathogen has spread rapidly, exhibiting tremendous morbidity and mortality to such an extent that the World Health Organization (WHO) officially declared the coronavirus disease 2019 (COVID-19) a pandemic on March 11, 2020. Since the first reported death in China on January 11, 2020, over 2.6 million cases and more than 188,000 deaths have been reported worldwide [1]. Dry cough is the most common symptom of COVID-19, with fever occurring in just half of patients [2]. The respiratory, renal, and cardiovascular systems are often affected. Some cases require specialized management in the ICU, with mechanical ventilatory support, with a mortality rate above 50% in those cases, as reported in Europe [3].

Identified risk factors for developing ARDS includes older age, male sex, hypertension, diabetes mellitus (DM), and chronic kidney disease (CKD) [4]. In our patient, type 1 DM, hypertension, and CKD secondary to IgG4 tubulointerstitial nephritis were the associated risk factors. Currently, COVID-19 caused by SARS-CoV-2 remains without definitive therapies. Some potential pharmaceutical targets have been identified but are still lacking proper validation through randomized clinical trials [5]. In this case report, we describe a young, immunosuppressed and critically ill female patient with previous Influenza B infection, which was then followed by SARS-CoV-2 infection in the following months.

## 2. Case Presentation

We describe the case of a 22-year-old Hispanic female, with a past medical history of biopsy-proven IgG4 tubulointerstitial nephritis on corticosteroids since December 2019, hypertension, and type 1 diabetes mellitus who was admitted twice, since the beginning of 2020, for severe acute hypoxic respiratory failure.

During her first admission in January, she presented with complaints of nonproductive cough and shortness of breath (SOB) and was diagnosed with multifocal pneumonia, secondary to Influenza B, diagnosed via PCR from a nasopharyngeal swab. She was treated with oseltamivir for 10 days and 3 doses of baloxavir marboxil for double influenza coverage, empiric ceftriaxone-azithromycin for 5 days for superimposed bacterial infection, and continued on sulfamethoxazole/trimethoprim for Pneumocystis prophylaxis. Hospitalization was complicated by hypoxic respiratory failure requiring endotracheal intubation and subsequent venous-venous extracorporeal membrane oxygenation (ECMO) for 5 days, prior to recovery and discharge, after a fourteen-day hospital course.

**Figure 1:**
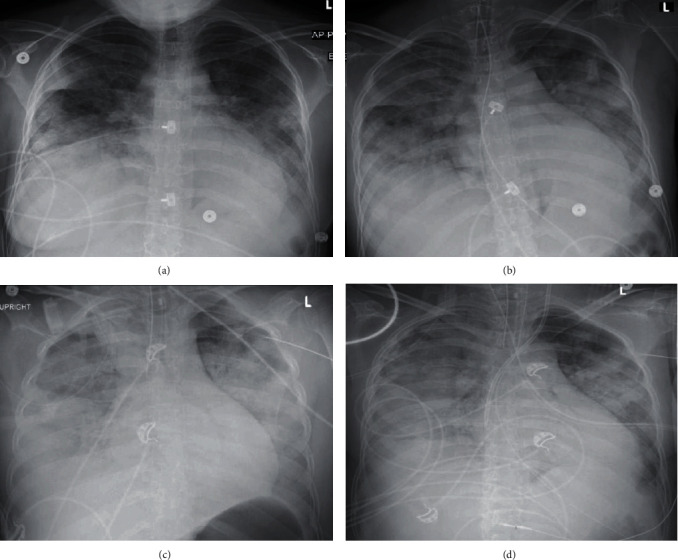
CXR with different infiltrates patterns during the initial 24 hours. (a) Initial CXR at the ED revealing bilateral pleural effusions with bibasilar consolidation, increased interstitial markings suggestive of bilateral pulmonary edema, and enlarged cardiac silhouette. (b) After endotracheal intubation with increased confluent airspace opacities throughout the mid-to-lower lungs, findings suggestive of worsening pulmonary edema vs. multifocal infectious process. (c) Findings with the tip of the endotracheal tube overlying the proximal right mainstem bronchus. Otherwise; the bilateral diffuse confluent airspace opacities are not significantly changed. Tube was retracted 2 cm. (d) Ten hours after initial CXR revealing stable cardiomegaly, persistent bilateral fluffy infiltrates and consolidation, and persistent obscuration of the right hemidiaphragm.

Two months after initial admission, the patient presented to the emergency department (ED) with one day of SOB, fever, chills, and dry cough. She developed progressive hypoxia requiring endotracheal intubation in the ED despite noninvasive mechanical ventilation. Sequential chest X-ray (CXR) during the first 24 hours did not correlate with the degree of hypoxemia (Figure 1). She was immediately transferred to the ICU, requiring vasopressors and ventilatory support, demanding constant adjustments to the ventilator settings. Early neuromuscular blockade was achieved with rocuronium for management of early severe adult respiratory distress syndrome (ARDS). She was empirically started on meropenem IV 1000 mg Q12H, azithromycin IV 500 mg daily, and hydroxychloroquine 200 mg Q12H, for empiric bacterial and COVID-19 pneumonia treatment, and continued on sulfamethoxazole/trimethoprim 800 mg/160 mg daily for Pneumocystis prophylaxis and prednisone 40 mg daily for IgG nephropathy. Infectious disease and nephrology were consulted. Initial and trended laboratory findings are further elucidated in (Table 1).

**Figure 2:**
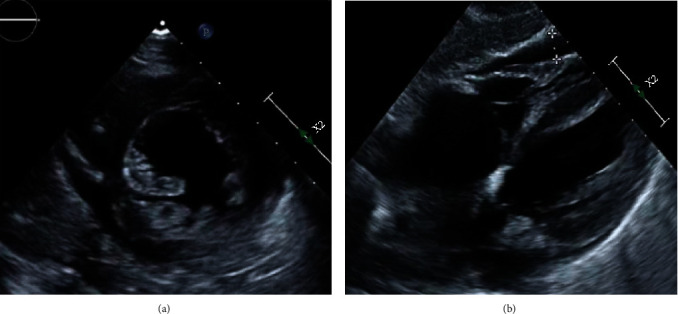
TTE showing a free-flowing pericardial effusion identified circumferentially to the heart. Mild decrease of her left ventricular systolic function, from 50% in January 2020 to 40-45%. (a) Parasternal short axis view. (b) Subxiphoid view.

On day one of ICU admission, the patient went into pulseless electrical activity (PEA) due to profound hypoxemia and severe acidemia, requiring advanced cardiac life support (ACLS), followed by hypothermic protocol for 24 hours, and initiation of Furosemide drip for volume overload in a setting of acute kidney injury (AKI). ECMO was deemed not appropriate at that time. Additionally, a transthoracic echocardiogram (TTE) performed the same day revealed reduced ejection fraction (EF) estimated at 40-45% and a new free-flowing pericardial effusion (Figure 2). Interestingly, the viral panel evidenced coinfection with the human coronavirus (HCoV) NL63 strain and the novel 2019 coronavirus SARS-CoV-2 via PCR from a nasopharyngeal swab.

As days progressed, she required hemodialysis support and therapeutic anticoagulation for suspected pulmonary embolism (PE). After seven days in the ICU, she spiked fevers along with worsening leukocytosis, and uptrending inflammatory markers, particularly ferritin, D-dimer, and LDH. She was restarted on antibiotics (vancomycin/piperacillin-tazobactam) for possible ventilator-associated pneumonia and received one dose of tocilizumab. Over the next days, her urine output increased and her PEEP and FiO2 requirements were weaned. By day 18, voriconazole was added due to increasing suspicion for superimposed invasive fungal infection, as the sputum cultures grew yeast and *β*-d-glucan levels were elevated.

Two days later, she was started on inhaled nitric oxide with decreasing doses of corticosteroids. After 25 days in the ICU, she achieved hemodynamically stability, without vasopressors. She improved to the point where she was arousable and able to engage in meaningful social interaction, when off sedation. A follow-up TTE showed recovered EF up to 55-60% and reduction of the pericardial effusion. Chronic hypoxic respiratory failure was attributed to extensive lung fibrosis, with lung transplant as the only definitive management. She underwent tracheostomy. Her urine output was fair while on IV furosemide, but her serum creatinine remained elevated without dialysis, indicating some lack of clearance. She underwent twenty-four-hour urine collection for creatinine clearance which shows markedly decreased kidney function with a creatinine clearance of 6 mL/min. A long-term dialysis catheter (permcath) was placed for continuity of hemodialysis sessions.

**Figure 3:**
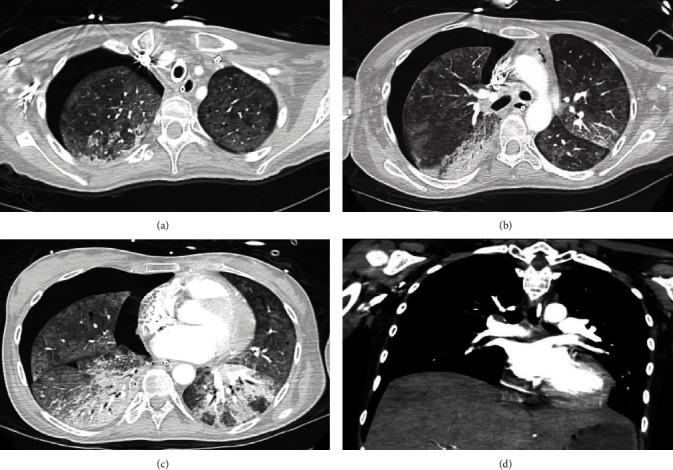
Computed tomography of the Chest. (a–c) Large right pneumothorax with small pneumomediastinum and mediastinal shift compatible with tension pneumothorax (unidentified on daily CXR). Diffuse ground-glass opacities with dense consolidations in both inferior lobes, early fibrosis in the right middle lobe, and multiple pneumatoceles. (d) Acute segmental pulmonary embolism is noted in the posterior segment of the right lower lobe.

**Figure 4:**
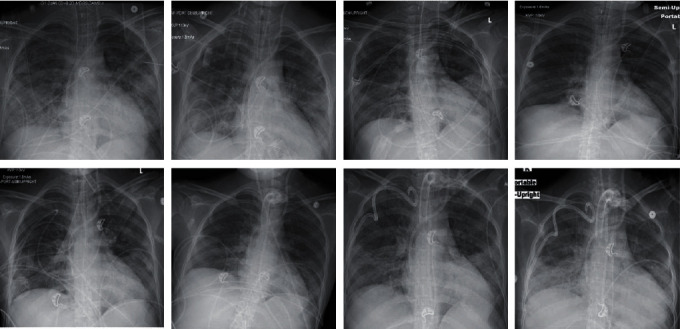
CXR progression until tracheostomy and chest tube insertion. Overall images were remarkable for stable appearance of subtle ill-defined hazy opacities throughout both mid to lower lungs. Infiltrates were always most accentuated in the left lung. No evidence of obvious pneumothorax or pneumomediastinum.

After 30 days, a computed tomography (CT) of the chest confirmed a segmental pulmonary embolism, along with a large right pneumothorax, small left pneumomediastinum, and leftward mediastinal shift (Figure 3), which were not observed in daily CXR (Figure 4). A thoracostomy tube was placed. The next day, however, she suffered multiple episodes of arrest with PEA, and despite multiple resuscitations, she perished.

## 3. Discussion

The SARS-CoV-2 has infected over 2.4 million people across 180 countries in four months, with a huge spectrum of clinical presentations, laboratory results, and imaging [6]. The virus invades cells by binding and crossing the cellular membrane via the angiotensin-converting enzyme 2 (ACE-2) receptor. This protein is found in high concentrations within the lungs and the heart [7, 8]. The most common complications of COVID-19 infection include ARDS, acute kidney injury, electrolyte disturbances, hypoproteinemia, and coagulation disorders. The median time from admission to the development of ARDS was two days in Wuhan, China [4].

Today, seven different coronavirus strains are known to infect humans including the HCoV-229E, HCoV-NL63, and HCoV-HKU1 [9, 10]. By itself, HCoV-NL63 infection has been described mostly in people <30 years [11–13]. This virus may have the ability to evade the human immune system, and thus patients with baseline immunosuppression are susceptible for more severe disease [13]. Coinfection with two different HCoV strains has not been commonly reported, but recently, a study limited to Northern California evidenced that 3.5% infected by SARS-CoV-2 were also positive for other types of coronavirus [14]. Further, superimposed invasive fungal infections have been reported in severe COVID-19, especially on those patients treated with tocilizumab who were concurrently taking immunosuppressive therapy, as it was the case in our patient.

Hospitalized patients with COVID-19 have a high prevalence of kidney disease, and this correlates with greater in-hospital mortality [15]. Decreased kidney function is usually temporal and likely related to profound hypoxia [16]. Avoidance of nephrotoxins to preserve remnant kidney function is indispensable. Furthermore, evidence in patients with DM and SARS-CoV-2 is still limited [17]; however, previous studies have described that individuals with diabetes are at risk of severe respiratory disease when infected with respiratory viruses [18].

Abnormal liver function panel, coagulopathies, and elevated D-dimer are common laboratory findings, arising suspicion for disseminated microthrombotic events and collateral pulmonary embolism [19]. Initiating empiric therapeutic anticoagulation is a topic of discussion, without clear criteria. However, recent evidence pointed to the types of patients who will receive the biggest benefits from anticoagulation [20, 21]. It should be started after carefully weighing the risks and benefits as was done in our young patient, who started therapeutic doses of heparin based on elevated D-dimer associated with increasing oxygen demands, prior to PE being confirmed.

Multiple medical interventions have emerged as possible therapeutic targets for SARS-CoV-2, such as corticosteroids, antibiotics, antiparasitics, antiretrovirals, and IL-6 inhibitors. Nevertheless, the safety and efficacy of these agents have not been properly validated through a rigorous randomized clinical trial. Regardless, several retrospective, observational, and open-label studies are now available, although with disparate results. One confusing area has been the use of corticosteroids, which are beneficial in critically ill patients, but their use has been debated in SARS-CoV-2. In our case, the patient was previously on prednisone for tubulointerstitial nephritis, and therapy was continued mostly to avoid adrenal insufficiency [22–24].

Hydroxychloroquine and azithromycin combination shows no clinical benefits in hospitalized patients with severe COVID-19, despite in vitro antiviral activity [25]. SARS-CoV-2 provokes a hyperinflammatory syndrome with fatal hypercytokinemia (including IL-6) and multiorgan failure [26]. Tocilizumab achieved benefits in isolated cases of SARS-CoV-2 infection, but its overall safety and efficacy are currently being analyzed [27, 28]. Our patient rapidly progressed to ARDS, which precluded a favorable clinical response to antimicrobial therapy. In retrospect, other medications that may have helped our patient include the antiparasitic ivermectin and the antiretroviral remdesivir [29, 30].

A new interesting proposition from Dr. Gattinoni et al. [31, 32] reflects the possibility of different types of lung pathology. Based on his experience in Northern Italy, he stated that only 20 to 30% of patients fulfilled the criteria for severe ARDS. They describe two alternative classifications of ARDS, one referred to as Type L, an unusual form of ARDS, characterized by low elastase, high lung compliance, low ventilation : perfusion ratio, low lung weight without significant edema, and low recruitability and a second referred as Type H, characterized by high elastase, low lung compliance, and significant right-to-left shunt, which is more consistent with standard ARDS criteria. Further, it has been hypothesized that SARS-CoV-2 Type L pneumonia may be related to the loss of regulation of alveoli perfusion and the loss of the hypoxic pulmonary vasoconstriction effect; the reason why it is feasible to think that inhaled nitric oxide may play a pivotal role. In the former guideline, a trial of inhaled pulmonary vasodilator as rescue therapy in patients with severe ARDS and persistent hypoxemia is suggested; however, its routine use is not recommended. In our case, the trial of nitric oxide improved the oxygenation [33].

We provided early neuromuscular blockade as this action provides a higher survival rate in early ARDS [34]. Additionally, strategies such as low TV, high PEEP, and prone position were used. Nevertheless, she developed a large pneumothorax and pneumomediastinum which were never visible on daily CXR, pointing in favor of sustained barotrauma. It is important to be aware of the damage associated with ventilatory support, due to the increased risk of mortality with some strategies [35]. Targeting a driving pressure-limited strategy provides better outcomes [36]. Physicians should be aware of unrecognizable pneumothorax at CXR as an associated damage for prolonged ventilatory support and barotrauma.

Our patient required ACLS on multiple occasions, with special measures taken, as healthcare workers have the highest risk of contracting SARS-CoV-2. New recommendations include reducing exposure, prioritizing oxygenation strategies with lower aerosolization risk, and considering the appropriateness of resuscitation, based on age, comorbidities, and severity of illness [37].

## 4. Conclusions

SARS-CoV-2 principally invades pneumocytes via the ACE-2 receptor, generating a significant inflammatory cascade that can render critical illness, associated with high mortality, in the form of ARDS. Coinfection with other respiratory pathogens and opportunistic infections are possible. SARS-CoV-2 infection can elicit different pathophysiological processes which may require multidisciplinary strategies. Of note, inhaled nitric oxide to increase alveolar perfusion should be considered in cases of poor response to “classic” ARDS management.

## Figures and Tables

**Table 1:** Laboratory test results.

Laboratory test	Reference values	Day 1	Day 3	Day 6	Day 9	Day 12	Day 15	Day 18	Day 21	Day 24	Day 25
White blood cells (×10^9^/L)	4.2-11	11.9	18.9	12.5	11.1	13.1	15	20.4	22.8	19.8	20.1
Absolute neutrophiles (×103)	1.8-7.7	11.1	18	11.8	9.8	12.2	13.7	19	20.1	17.4	16.8
Absolute lymphocytes (×10^3^)	1.4-4.0	0.5	0.6	0.5	0.9	1	0.6	1	0.7	0.6	0.4
C-reactive protein (mg/dL)	<1		30.1	17.8		10.4	3.4	8.2		1	0.5
Ferritin (ng/mL)	8-252	711	14,188	7,350	4,844	5,127	2,736	2,868	2,927	4,720	5,607
LDH (U/L)	82-240	341	693	862	811	683		715	515		
Procalcitonin (ng/mL)	<0.1	11.32		3.01	3.89	1.22		1.63			
Interleukin-6 (pg/mL)	≤5			16							
Troponin 1 (ng/mL)	<0.05	0.1	0.46	0.07							
NT-proBNP (pg/mL)	<451	30,860		18,041		22,398					
Creatinine (mg/dL)	0.51-0.95	4.87	5.09	5.92	3.73	3.29	4.3	3.04	3.11	2.85	3.01
eGFR (mL/min/1.73 m^3^)	>90	12	11	9	16	19	14	21	20	23	21
D-dimer (*μ*g/mL)	<0.57	1.08			14.52	8.23	5.85			3.74	4.42
AST (U/L)	<38	22		59		45	45	82	51	480	264
ALT (U/L)	<64	43		75		17	21	49	72	368	373
Total bilirubin (mg/dL)	0.2-1.0	0.2				0.2	0.2	0.3	0.3	3	4.2
Creatine kinase (U/L)	26-192					43					
Lactate (mmol/L)	0.0-2.0	2.6				1.4					
Triglycerides (mg/dL)	<115			450		315		166	246	672	
pH	7.35-7.45	7.31	7.32	7.36	7.37	7.37	7.33	7.37	7.38	7.38	7.26
PaCO2 (mmHg)	32-45	31	37	33	46	50	47	46	41	34	37
PaO2 (mmHg)	83-108	66	97	72	64	73	60	77	76	63	83
PaO2 to FiO2 ratio	300-500	66	162	120	128	122	86	110	126	157	165
PEEP		12	12	10	12	15	15	18	18	12	10
FiO2 (%)		100	60	60	50	60	70	70	60	40	50
(1,3)-beta-D-glucan (pg/mL)								138(+)			
HCoV-NL63		+									
Novel 2019 SARS-CoV-2		+									

eGFR: estimated glomerular filtration rate; AST: aspartate aminotransferase; ALT: alanine aminotransferase; LDH: lactate dehydrogenase; PaCO_2_: partial pressure of carbon dioxide; PaO_2_: partial pressure of oxygen; FiO_2_: fraction of inspired oxygen; O_2_ sat: oxygen saturation; PEEP: positive end-expiratory pressure.

## Data Availability

Data availability is not declared.

## Abbreviations

ACLS: Advanced cardiac life support
ARDS: Adult respiratory distress syndrome
COVID-19: Coronavirus disease 2019
CXR: Chest X-ray
DM: Diabetes mellitus
ECMO: Extracorporeal membrane oxygenation
NMB: Neuromuscular blockade
ROSC: Return of spontaneous circulation
SARS-CoV-2: Severe acute respiratory syndrome coronavirus 2
SOB: Shortness of breath
TTE: Transthoracic echocardiogram.
